# Matta’s criteria may be useful for evaluating and predicting the reduction quality of simultaneous acetabular and ipsilateral pelvic ring fractures

**DOI:** 10.1186/s12891-021-04441-z

**Published:** 2021-06-14

**Authors:** Yi-Hsun Yu, Chang-Heng Liu, Yung-Heng Hsu, Ying-Chao Chou, I-Jung Chen, Chi-Chuan Wu

**Affiliations:** Department of Orthopedic Surgery, Musculoskeletal Research Center, Chang Gung Memorial Hospital, Chang Gung University, 5, Fu-Hsin St. Kweishan, 33302 Tao-Yuan, Taiwan

**Keywords:** Pelvic fracture, Acetabular fracture, Simultaneous fracture, Matta criteria, Reduction quality

## Abstract

**Background:**

Although the incidence, types, and radiological outcomes of simultaneous ipsilateral pelvic ring and acetabular fractures have been reported, there have been no reports on factors that may affect the quality of acetabular fracture reduction. Here, we evaluate the radiological outcomes of patients treated for simultaneous ipsilateral pelvic and acetabular fractures and analyze the factors that affect the quality of acetabular fracture reduction.

**Methods:**

We conducted a retrospective review of patients treated for simultaneous ipsilateral pelvic ring and acetabular fractures between 2016 and 2020. Factors that may predict inadequate reduction of the acetabular fracture were analyzed.

**Results:**

Data from 27 hips of 26 patients were collected. AO B2.2 and anterior columnar fractures were the most common types of pelvic ring and acetabular fractures, respectively. Univariate analysis revealed that Matta’s criteria for pelvic ring fracture may be useful for predicting fair to poor quality of acetabular fracture reduction on X-rays. Furthermore, associated fractures identified by Letournel’s classification system on computed tomography may be predictive of greater step-offs.

**Conclusions:**

Associated fractures identified via Letournel’s classification may contribute to inadequate reduction of acetabular fractures. Matta’s criteria for pelvic ring fractures may also be useful for predicting the risk of inadequate reduction of the acetabulum on X-ray scans. These findings may be assessed intraoperatively by fluoroscopy before beginning osteosynthesis for acetabular fractures.

## Background

Successful treatment of pelvic ring and acetabular fractures remains challenging for orthopedic surgeons, especially for multi-planar unstable pelvic and complex acetabular fractures [1–4]. Unstable pelvic ring and acetabular fractures are usually caused by high energy traumas, such as high-speed motor vehicle accidents, falls from height, and crush injuries. The treatment strategies include surgical restoration of the biomechanics of the pelvic ring and the congruency of the hip joint aim, which can achieve good outcomes and allow quicker return of the patient to activities of daily living.

The goals of osteosynthesis for pelvic ring fracture are to restore bone continuity and symmetry as well as biomechanical stability. Anatomical reduction of the pelvic fracture is crucial since an unstable or asymmetric pelvic ring may lead to chronic pain and long-term morbidity [5–7]. Similarly, the goals of osteosynthesis for acetabular fracture are anatomical reduction and rigid fixation, and thereby restoration of joint congruency. Residual non-anatomical reduction following acetabular fractures may lead to poor surgical outcomes and early onset post-traumatic osteoarthritis [7–10].

Since satisfactory reduction is similarly required for pelvic ring and acetabulum fractures in patients with both, the reduction sequence is of utmost importance. Several previous studies on combined pelvic ring and acetabular fractures have focused on the incidence, treatment protocol, treatment sequence, and radiological outcomes [4, 7, 11–13]. In general, there has been a consensus on posterior pelvic ring reduction and fixation before osteosynthesis for acetabular fractures. However, there are no known factors for evaluating the reduction quality after pelvic ring fractures and before osteosynthesis for acetabular fractures. Such factors could be useful for predicting poor reduction quality. Therefore, the current study aimed to (1) evaluate the radiological outcomes of patients with simultaneous ipsilateral pelvic ring and acetabular fractures treated at a single trauma center and (2) identify factors associated with non-anatomical reduction of the acetabulum.

## Methods

We performed an IRB-approved retrospective review of the medical records and images of patients with simultaneous ipsilateral pelvic ring and acetabular fractures (2016–2020) treated at a level-one trauma center. Age, sex, injury mechanism, injury severity score (ISS), and surgical details were recorded. The inclusion criteria of the current study were as follows: (1) patients with simultaneous pelvic ring and acetabular fractures of the ipsilateral body, (2) age > 18 years, (3) patients who underwent osteosynthesis for the pelvic ring and acetabular fractures, and (4) complete clinical and imaging follow-ups. Patients were excluded for the following: (1) injuries of the pelvic ring and acetabulum located at opposite sites, (2) conservative treatment for either pelvic ring or acetabulum fracture, or (3) incomplete radiological follow-up.

All patients were treated by one senior surgeon. After the patients were resuscitated and medically optimized, osteosynthesis was performed as soon as possible. All patients underwent complete radiological examinations, including X-ray (anterior-posterior [AP], inlet, outlet, iliac oblique, and obturator oblique views) and computed tomography (CT) scans. Operative indications for pelvic ring fractures were significant displacement (> 2 cm) in a stable pelvic fracture, rotational instability, and global (rotational + vertical) instability. Similarly, the surgical indications for acetabular fracture were displacement of components (wall, column, or both) by > 2 mm, incongruent hip joint, intra-articular osteochondral fragments, and a persistently subluxated or dislocated hip joint.

Various surgical approaches were utilized according to the fracture patterns. Generally, the reduction sequence was initially started from the posterior pelvic ring for spinopelvic dissociation, crescent fractures, sacral fractures, and sacroiliac joint diastasis. Once the pelvic fracture or dislocation was reduced and fixed, either temporarily or permanently, the osteosynthesis was changed to reduce and fix the acetabular fractures. A single or two or more surgical approaches (simultaneous or sequential) were performed, depending on the presentation of the fractures. All of the patients underwent postoperative image examinations, including X-ray (AP, inlet, outlet, and 2 Judet views) and CT scans, to evaluate the reduction quality. We used the picture archiving and communication system to adjust the magnification of the area of interest. All radiographs were assessed using the same approach.

Several classifications and parameters for evaluation of imaging outcomes were adopted in this study. We used the AO classification for the pelvic ring fracture [14] and Letournel’s classification for the acetabular fractures [5]. The radiological outcomes of the pelvic ring injury were determined using (1) Matta’s criteria [14, 15], (2) the inlet and outlet ratio proposed by Sagi et al. [16], and (3) Henderson’s criteria [17]. Lefaivre’s method was used to evaluate pelvic vertical and rotational displacement and the symmetry of the pelvis [18]. The acetabular fracture reduction quality was evaluated on AP and 2 Judet views on X-ray scans using Matta’s criteria [19]. In addition, the quality was also evaluated by postoperative CT scans, including axial, coronal, and sagittal planes, to determine the maximum residual step-off after osteosynthesis. All of the images were interpreted by 3 surgeons. For Matta’s criteria, if the surgeons had similar interpretations, the mean score was recorded. If there was a difference in interpretation but 2 surgeons had similar interpretations, the differences were overlooked and the score was reported. However, if all surgeons provided different interpretations, another senior surgeon (Y.-H, Y.) interpreted the images to determine the final score.

Continuous variables are reported as the mean and standard variation. Categorical variables are reported as the number of patients per variable. The distribution of the continuous variables was examined using the Kolmogorov-Smirnov test and the Shapiro-Wilk test. Univariate and multivariate analyses were conducted to determine the potential findings on X-ray and CT images that may contribute to poor acetabulum reduction quality. The univariate analysis was performed using Fisher’s exact test, multiple logistic regression, general linear models, and the Kruskal-Wallis and Mann-Whitney U tests. The multivariate analysis was performed using a general linear model and multiple logistic regression. Inter-observer reliability was also calculated and reported as Cronbach’s alpha.

## Results

During the study period, 26 pelvises and 27 hips of 26 eligible patients were included in this study. There were 17 men and 9 women with a mean age of 47.8 ± 14.8 years. The mean ISS was 16.7 ± 8.6 (median: 17, interquartile range: 12). The most common fracture type was AO B2.2 for the pelvis, and anterior column for the acetabulum. Nineteen pelvic fractures were defined as having rotational instability and 7 as having global instability. Fourteen acetabular fractures were classified as elementary and 13 as associated fractures. There were no isolated posterior wall, anterior wall, or posterior column fractures in this cohort. The demographic data of the patients are shown in Table 1.

**Table 1:** Demographic data of patients with simultaneous ipsilateral pelvic and acetabular fractures

Patient number	26
Sex	
Male	17
Female	9
Age (year-old)	47.89 ± 14.82
Trauma Mechanism	
Motorbike accident	11
Fall from height (>6 meter)	11
Car accident	3
Pedestrian injury	1
Fracture classification	
Pelvis (AO classification)	
A1.1	1
B2.1	2
B2.2	10
B2.3	3
B3.2	1
B3.3	2
C1.3	4
C3.1	2
C3.3	2
Acetabulum (Letournal classification)	
Anterior column	10
Transverse	4
Transverse + posterior wall	1
T-shape	1
Anterior column plus	4
hemi-transverse	
Associated both columns	5
T-shape plus posterior wall ^a^	2
Sacral fracture	
Rotational instability	7
Rotational + Vertical instability	6
Sacral-iliac joint diastasis	14
Injury severity score	16.7 ± 8.6 (median: 17, interquartile range: 12)
Follow up (mons)	14.7 ± 8.90 (range: 3 – 43)
Post-traumatic osteoarthritis (THA)	2 hips (in one patient)

^a^This type of fracture was not included in Letournal’s classification

Ten patients underwent a single approach for simultaneous reduction and fixation of the pelvis and acetabulum, and 16 underwent simultaneous or sequential approaches. A variety of approaches were devised and applied to the patients according to the fracture types and combined injuries/fractures of the chest wall, abdominal cavity, and lower extremity. The perioperative data are shown in Table 2.

**Table 2:** Periopriative data patients with simultaneous ipsilateral pelvic and acetabular fractures

Operation duration (mins)	236.67 ± 86.86
Estimated blood loss (mL)	783.33 ± 400.17
Number of operations	
Single approach	10
Two or more approaches	16
Approach Sequence	
Anterior first	19
Posterior first	8
Approaches	
Ilioinginal	8
Anterior intrapelvis	13
Kocher-Lagenbeck	5
Gibson + trochanteric osteotomy	2
Iliosacral or trans-iliac trans-sacral screws	5
Open reduction for sacroiliac joint	3
Pararectus	4
Spinopelvic osteosynthesis	3

To determine the postoperative quality of the pelvis and acetabulum reduction, several diagnostic criteria were adopted (Table 3). The X-ray examinations revealed an average discrepancy in rotational instability of 9 mm for the pelvic ring. Residual step-off after osteosynthesis was also detected via CT, with the widest step-off distance (2.63 ± 2.57 mm) observed on the coronal view rather than the axial and sagittal views. The inter-observer reliability was excellent despite the use of Matta’s criteria for predicting the quality of the pelvic reduction (Table 3).

**Table 3:** Reduction quality evaluations of the pelvis and acetabulum and inter-observer reliability

Pelvis			Acetabulum		
Parameters	Outcome	Cronbach's Alpha	Parameters	Outcome	Cronbach's Alpha
Matta’s criteria		0.658	Matta’s criteria (X ray)		
Excellent	25		Anteroposterior view		
Good	1				
Fair	0				
Poor	0		Excellent	17	0.988
			Fair	8	
			Poor	2	
			Iliac oblique view		
			Excellent	17	0.972
			Fair	9	
			Poor	1	
			Obturator oblique view		
			Excellent	17	0.985
			Fair	5	
			Poor	5	
Sagi			CT scan (residual step-off, mm)		
Inlet ratio	0.96 ± 0.06	0.921	Axial	1.62 ± 1.56	0.988
Outlet ratio	1.02 ± 0.17	0.973	Coronal	2.63 ± 2.57	0.993
			Sagittal	1.27 ± 1.50	0.998
Henderson’s criteria^a^	-3.28 ± 10.24	-			
Lefaivre’s method^a^	-9.10 ± 11.15	-			

^a^The value was calculated by (lesion hemipelvis) minus (intact hemipelvis)

Among the examined factors, the univariate analysis showed that associated fractures (Letournel’s classification) and quality of the pelvic reduction, as evaluated by Matta’s criteria, may contribute to inadequate reduction of the acetabulum on the AP view of iliac obturator X-ray scans (Table 4). Furthermore, associated fractures of the acetabulum were the only predictive factor on axial and sagittal CT views. However, no single X-ray or CT factor that affects the quality of the acetabulum reduction was identified in the multivariate analysis.

**Table 4:** Univariate analysis of factors involving reduction quality of the acetabulum on X rays (anteroposterior, iliac oblique, and obturator oblique views) and CT scan (axial, coronal and sagittal views)

Selected factors	X ray			CT scan		
	*P* value			*P* value		
	AP	Iliac oblique	Obturator oblique	Axial	Coronal	Sagittal
Classification of pelvic fracture (AO)	1	0.159	0.37	0.788	0.658	0.297
SI joint diastasis	0.327	0.067	0.114	0.322	0.519	0.217
Sacral fracture	0.931	0.456	0.763	0.639	0.702	0.187
Classification of acetabular fracture (Letournel)	0.001^*^	0.007	0.168	0.002*	0.059	0.020*
Sequence of surgical approaches	0.599	0.748	0.511	0.447	0.680	0.680
Reduction quality of pelvis (Matta)	0.011*	0.030*	0.335	0.087	0.708	0.173
Reduction quality of pelvis (inlet ratio)	0.412	0.941	0.414	0.107	0.650	0.407
Reduction quality or pelvis (outlet ratio)	0.862	0.823	0.470	0.985	0.354	0.209
Vertical reduction quality or pelvis	0.620	0.291	0.479	0.377	0.481	0.174
Rotational reduction quality of pelvis	0.966	0.177	0.130	0.109	0.159	0.291

*Statistical significance

## Discussion

The current study examined the radiological results of simultaneous surgical treatment of ipsilateral pelvic ring and acetabular fractures to evaluate the factors that may contribute to inadequately reduced acetabular fractures. The results revealed that the most common fracture type was B2.2 for pelvic ring fractures and anterior column for acetabular fractures. Additionally, the univariate analysis demonstrated that associated acetabular fractures and quality of the pelvic reduction, according to Matta’s criteria, were the two factors that affected the reduction quality of subsequent acetabular fractures. However, no significant factors were identified in the multivariate analysis.

Anatomical reduction to minimize the risk for post-traumatic osteoarthritis is the main goal of osteosynthesis for acetabular fractures. Tannast et al. reviewed a series of 816 patients with acetabular fractures and followed them for 2–20 years [8]. They showed that non-anatomical fracture reduction was the most significant factor associated with the requirement for total hip arthroplasty. Additionally, most studies that reported the outcomes after acetabular fractures have indicated the importance of anatomical fracture reduction [8–10, 19, 20]. There are several factors that might affect reduction of acetabular fractures, including complexity of the fracture pattern. Thirteen acetabular fractures (48.1 %) were classified as associated fractures using Letournel’s classification. Among the 13 associated acetabular fracture patterns, 10 were graded as being of fair to poor reduction quality on AP iliac, oblique, or obturator oblique view X-ray. Additionally, there were greater step-offs on CT scans for the associated fracture patterns. Therefore, our results show that the classification might lead to inadequate reductions of acetabular fractures.

However, the acetabulum is part of the pelvis; therefore, in cases of pelvic ring fracture without sufficient reduction, the reduction of the acetabulum may not be adequate. Suzuki et al. confirmed that the initial adequate reduction of the posterior pelvic lesion is necessary to obtain optimal reduction of the acetabulum [4]. Since accurate reduction of the posterior pelvic ring is key to anatomical reduction of the acetabular fracture, the sequences of fracture reduction and fixation may be critical. Although this study consisted of 19 patients undergoing treatment by anterior approaches (ilioinguinal, anterior intrapelvic, and pararectus), the principles of the reduction sequence did not differ. These approaches mostly aimed to reduce and fix anterior lesions; however, posterior pelvic ring injures, such as crescent fracture, sacral fractures, and sacroiliac joint diastasis, can also be managed using these approaches. On the other hand, posterior approaches were still necessary when a displaced posterior pelvic ring existed, and should be performed prior to anterior approaches. There were three cases of spinopelvic osteosynthesis and three cases of open reduction and fixation to address posterior sacroiliac joint injuries. These six patients also underwent subsequent anterior approaches for treatment of the acetabular fractures.

The reduction of the posterior pelvic ring is crucial in obtaining satisfactory radiological results; however, there has been no discussion on how to optimally evaluate the pelvic reduction quality before beginning osteosynthesis of the acetabulum. Our study shows that Matta’s criteria may be useful in evaluating the reduction of the acetabulum. Since fluoroscopy is the most commonly used tool for intraoperative evaluation of fracture reduction, Matta’s criteria can be used before osteosynthesis for acetabular fractures to determine whether the reduction should be more accurate before proceeding with the osteosynthesis. This method may be applied intraoperatively to ensure the quality of the subsequent reduction of the acetabulum.

There have been similar reports of combined pelvic ring and acetabular fractures [4, 7, 11, 12, 21]. According to previous findings, the most common fracture type of the pelvis in similar cohorts was the lateral compression type [4, 7, 21]. However, Osgood et al. found that the AP compression type was the most common in their cohort [11]. In our study, the most common fracture type was the lateral compression type. Because the most common type of pelvic fracture in our study was B2.2 (lateral compression type), the injury force was probably applied directly and laterally towards the greater trochanter of the femur, which would be similar to the injury force resulting in anterior column fractures of the acetabulum [22], the most common type of acetabular fractures in our study. Therefore, we observed similar fracture types from injuries to the pelvis and acetabulum, consistent with previous studies.

Although we made efforts to avoid bias, this study had some limitations. First, this study included a relatively small number of patients, which might result in statistical bias. However, similar to previous studies, the incidence of simultaneous pelvic and acetabular fractures was low, and the number of enrolled patients was naturally limited. Second, although one of the major findings of this study was the proposal to apply Matta’s criteria for intraoperative assessment of the pelvic ring, this was evaluated using postoperative images. The actual intraoperative usefulness of Matta’s criteria in predicting the quality of acetabular fracture reduction in this cohort should be determined in a future study. However, the strength of our study includes the fact that the patients were all treated by a single surgeon at a single institute with a similar treatment protocol. All patients were followed up with complete postoperative X-ray and CT evaluations, and 3 independent examiners interpreted the imaging findings with excellent inter-observer reliability. Therefore, the data obtained were reliable and convincing. Owing to the aforementioned limitations, a meta-analysis should be conducted in the future to thoroughly investigate this specific group of patients.

## Conclusions

The results of the current study demonstrated that Letournel’s classification for associated fractures of the acetabulum, and Matta’s criteria for pelvic ring injuries, might be useful in predicting the quality of acetabular fracture reduction in patients with simultaneous ipsilateral pelvic ring and acetabular fractures. The findings could be applied preoperatively (Letournel’s classification) and intraoperatively (Matta’s criteria) before starting osteosynthesis of the acetabulum.

## Data Availability

All data generated or analyzed during this study are included in this published article. The datasets used and/or analyzed during the current study are available from the corresponding author on reasonable request.

## Abbreviations

AP: Anteroposterior
CT: Computed tomography
